# Transplantation of human fetal pancreatic progenitor cells ameliorates renal injury in streptozotocin-induced diabetic nephropathy

**DOI:** 10.1186/s12967-017-1253-1

**Published:** 2017-06-27

**Authors:** Yongwei Jiang, Wenjian Zhang, Shiqing Xu, Hua Lin, Weiguo Sui, Honglin Liu, Liang Peng, Qing Fang, Li Chen, Jinning Lou

**Affiliations:** 1 Department of Laboratory Medicine, China-Japan Friendship Hospital, Beijing, 100029 China; 2 Institute of Clinical Medical Sciences, China-Japan Friendship Hospital, No. 2 Yinghua East Street, Chaoyang District, Beijing, 100029 People’s Republic of China; 3 Department of Gynecology and Obstetrics, China-Japan Friendship Hospital, Beijing, 100029 China; 4 First Kidney Transplantation Hemopurification Center of Chinese PLA, 181st Hospital of Chinese People’s Liberation Army, Guilin, 541002 China; 5 Department of Endocrinology, Qilu Hospital, Shandong University, Jinan, 250012 Shandong China

**Keywords:** Diabetic nephropathy, Human fetal stem cell, Cell transplantation, Mechanism

## Abstract

**Background:**

Diabetic nephropathy (DN) is a severe complication of diabetes mellitus (DM). Pancreas or islet transplantation has been reported to prevent the development of DN lesions and ameliorate or reverse existing glomerular lesions in animal models. Shortage of pancreas donor is a severe problem. Islets derived from stem cells may offer a potential solution to this problem.

**Objective:**

To evaluate the effect of stem cell-derived islet transplantation on DN in a rat model of streptozotocin-induced DM.

**Methods:**

Pancreatic progenitor cells were isolated from aborted fetuses of 8 weeks of gestation. And islets were prepared by suspension culture after a differentiation of progenitor cells in medium containing glucagon-like peptide-1 (Glp-1) and nicotinamide. Then islets were transplanted into the liver of diabetic rats via portal vein. Blood glucose, urinary volume, 24 h urinary protein and urinary albumin were measured once biweekly for 16 weeks. Graft survival was evaluated by monitoring human C-peptide level in rat sera and by immunohistochemical staining for human mitochondrial antigen and human C-peptide in liver tissue. The effect of progenitor-derived islets on filtration membrane was examined by electron microscopy and real-time polymerase chain reaction (PCR). Immunohistochemical staining, real-time PCR and western blot were employed for detecting fibronectin, protein kinase C beta (PKCβ), protein kinase A (PKA), inducible nitric oxide synthase (iNOS) and superoxide dismutase (SOD).

**Results:**

Islet-like clusters derived from 8th gestational-week human fetal pancreatic progenitors survived in rat liver. And elevated serum level of human C-peptide was detected. Blood glucose, 24 h urinary protein and urinary albumin were lower in progenitor cell group than those in DN or insulin treatment group. Glomerular basement membrane thickness and fibronectin accumulation decreased significantly while podocytes improved morphologically in progenitor cell group. Furthermore, receptor of advanced glycation end products and PKCβ became down-regulated whereas PKA up-regulated by progenitor cell-derived islets. And iNOS rose while SOD declined.

**Conclusions:**

DN may be reversed by transplantation of human fetal pancreatic progenitor cell-derived islets. And fetal pancreatic progenitor cells offer potential resources for cell replacement therapy.

**Electronic supplementary material:**

The online version of this article (doi:10.1186/s12967-017-1253-1) contains supplementary material, which is available to authorized users.

## Background

As a major risk factor for renal failure in patients with end-stage renal disease [1], diabetic nephropathy (DN) often leads to diabetic mortality. The occurrence and development of DN might involve complex pathophysiological interactions of inflammatory, metabolic and hemodynamic factors [2]. All these factors induce injuries of glomeruli, tubular epithelial cells, interstitial fibroblasts and vascular endothelial cells. Recent advances showed that oxidative stress played key roles in the pathogenesis of DN. Glucose-dependent pathways of advanced glycation end products (AGEs) were essential in the development of diabetic nephropathy. Accumulation of enhanced AGEs in kidney could directly modulate the expression of key components of renin-angiotensin system [3]. And AGEs interacted with their corresponding receptors (RAGE) in glomerular endothelial cells for up-regulating protein kinase C (PKC), suppressing protein kinase A (PKA) and activating oxidative stress response [4], a salient feature of microangiopathy in DN [5]. Also using PKC inhibitor or PKA agonist may arrest the onset and progression of DN.

Strict controls of glucose level and blood pressure have remained standard treatment for DN [6, 7]. However, the efficacy has been unsatisfactory. Recent studies confirmed that pancreatic and islet transplantation significantly reduced the risks of diabetic macrovascular complications and effectively reversed diabetic microvasculopathy [8–10]. However, due to an acute shortage of pancreas donor, pancreas and islet transplantation failed to meet clinical demands, greatly limiting its clinical application. Various biologically active products secreted by pancreatic islet cells, such as C-peptide and glucagon-like peptide 1 (GLP-1), have been efficacious for diabetic vasculopathy. With the potentials of self-renewal and cellular differentiation, stem cell technology featuring in vitro proliferation and directional differentiation for preparing functional islets might increase the supply of islet cells [11, 12].

Stem cells derived from early human embryonic pancreas had strong capacities of in vitro proliferation and directional differentiation. Specifically, human embryonic pancreas-derived progenitor cells were utilized for preparing progenitor cell-derived islets via induced directional differentiation. The progenitor cell-derived islets were transplanted into liver for evaluating treatment efficacy and elucidating mechanisms in DN rats. Our work might provide preliminary evidence for treating DN with progenitor cell-derived islets.

## Methods

### Animal modeling

The study protocol was approved by the Animal Ethics Committee of China-Japan Friendship Hospital. And 8-week-old male Wistar rats (250–300 g) were purchased from Beijing Vital River Laboratory Animal Technology Co., Ltd. (Beijing, China) and were allowed to adapt to the housing environment and diet for 1 week. Then the diabetic model was induced by administration of 12 g/L streptozocin (freshly diluted with 0.1 mol/L of citrate buffer at pH 4.5) intraperitoneally (30 mg/kg) twice within 1 week interval. Blood glucose of caudal vein >16.7 mmol/L for 3 consecutive days after second STZ-injection was considered as a standard of diabetic model. Successful diabetic modeling was confirmed if blood glucose exceeded 16.7 mmol/L for 3 consecutive days. Diabetic animals were fed with a normal diet for 6 consecutive months until an onset of severe kidney disease. Successful modeling of DN was confirmed if 24 h urinary output was >twofolds, urinary albumin >50-folds and urinary protein >threefolds in DN rats as compared to normal control rats. DN animals were divided randomly into three groups of progenitor cell (n = 6, transplantation of progenitor cell-derived islets into liver via portal vein), insulin-treated (n = 6, 2.5 IU/day insulin glargine, sc) and DN (n = 6, no treatment). And healthy Wistar rats of the same age were used as normal controls.

### Expansion and culturing of human fetal pancreatic progenitor cells

The study protocol was approved by the Clinical Research Ethics Committee of our hospital. Progenitor cells were isolated from aborted human fetal pancreas at gestational week 8. And human fetal pancreases were harvested after obtaining informed patient consents. As previously described [13], fetal pancreases were digested with collagenase XI at 37 °C for 15 min. Islet-like tissue rich in endocrine progenitor cells were collected and cultured in DMEM/F12 containing basic fibroblast growth factor, epidermal growth factor, leukemia inhibitor factor (Peprotech, NJ, USA) and 5% fetal bovine serum for stem cells.

Cells were expanded in growth factor enriched medium for about 30–45 days and the cells between passages 6–9 were used in this study. The marker expression of pancreatic endocrine progenitor cells was evaluated by immunofluorescent staining. The progenitor cells were differentiated for 3 weeks in culture medium of M199 containing 15% fetal bovine serum, glp-1 (10 nM) and nicotinamide (5 mM). The endocrine hormones of insulin and glucagon were measured by immunofluorescent staining. And the insulin release upon glucose stimulation was measured by enzyme-linked immunosorbent assay (ELISA).

### Transplantation of progenitor cell-derived islets

Differentiated cells were re-suspended and cultured overnight for inducing islet-like clusters. Progenitor cell-derived islet suspension was slowly injected into liver via portal vein (approximately 1000 islets equivalent per animal). Rats in the “Insulin Group” received 2.5 IU/day insulin glargine (sc).

### Functional assessments of progenitor cells in vivo

For glucose monitoring via tail vein, animals were placed into metabolic cages (Suzhou Fengshi Laboratory Animal Equipment Co., Ltd, Jiangsu, China) before and every 2 weeks after transplantation. A 24-h urinary measurement was made for each animal of each group. Urinary albumin was measured by ELISA (Assay Max Rat Albumin ELISA kit, Gentaur, Belgium) and urinary protein by BCA assay (BCA protein assay kit, Beyotime, Shanghai, China).

At week 16 post-transplantation, the serum level of human C-peptide level was measured by ELISA (DRG International Inc., NY, USA).

### Evaluations of cellular immunogenicity

#### Expression of HLA molecules

HLA classes I & II molecules on progenitor cells were detected by flow cytometry. In brief, single cell solution were blocked with 0.1% BSA in PBS, and then incubated with mouse anti-HLA classes I & II antibodies at 4 °C for 40 min, washed thrice with 0.1% BSA/PBS and incubated with Alexa488-conjugated donkey anti-mouse IgG respectively at 4 °C for 30 min. Fluorescence was detected by flow cytometry (Beckman coulter, CA, USA).

#### Activation of lymphocyte by progenitor cells

A total of 5 × 10^6^ progenitor cells undergone frozen-thaw thrice and then ultrasonicated for cell lysate. Rat lymphocytes were isolated by Ficoll, seeded in 24-well plate at a density of 1 × 10^6^/well and incubated with cell lysate for 24 or 48 h. PHA (10 μg/mL) and PMA (10 ng/mL) were used as positive controls. Supernatant was collected for measuring IL-2 concentration by ELISA (R&D, MN, USA).

### Histopathological and immunohistochemical stains

Dissected liver and kidney tissues were fixed for 48 h in 10% neutral buffered formalin and followed by conventional tissue processing and paraffin-embedding. Each paraffin-embedded sample was sectioned into 3-µm thick slices and stained by hematoxylin & eosin (H&E). And kidney sections were stained with periodic acid-Schiff (PAS) reagent. For immunohistochemical staining, liver sections were incubated with primary anti-human mitochondrial antigen, anti-human C-peptide and anti-human glucagon antibodies while kidney sections with primary anti-fibronectin (anti-FN), anti-RAGE, anti-PKC β, anti-PKA, anti-iNOS and anti-SOD1 antibodies. Subsequent incubation was made with corresponding horseradish peroxidase (HRP)-conjugated secondary antibodies and staining with 3,3′-diaminobenzidine (DAB) peroxidase substrate solution.

### Transmission electron microscopy

Renal cortex was fixed for 1 h at room temperature in 2.5% glutaraldehyde (prepared in 0.1 M phosphate buffer, pH 7.4). Specimens were processed routinely for electron microscopy. Morphology of glomerular podocytes was assessed under a TEM JEO1010. And the thickness of glomerular basement membrane (GBM) was measured and averaged (n > 25).

### Real-time polymerase chain reaction

Total RNA from isolated glomeruli were extracted with RNAeasy Mini (Qiagen, Germany). Two micrograms of total RNA template, Oligo dT primer and AMV reverse transcriptase (Invitrogen, Thermo Fisher Scientific Co., USA) were used for synthesizing first-strand cDNA. Real-time fluorescent quantitative polymerase chain reaction (PCR) were performed with SYBR green PCR reagent kit (TOYOBO, Osaka, Japan) on Applied Biosystems 7300 Real-Time PCR System (Life Technologies Corporation, Carlsbad, CA). And β-actin was utilized as a house keeping gene for normalizing mRNA expression using the 2^−ΔΔCt^ formula.

### Western blot

Glomerular protein was extracted with SDS protein lysate (KeyGEN, Nanjing, China), separated by sodium dodecyl sulfate–polyacrylamide gel electrophoresis (SDS-PAGE) and transferred onto a PVDF membrane. PVDF membrane was blocked with 5% skim milk containing 0.05% Tween-20 and incubated with individual primary antibodies, including anti-FN (Santa Cruz Biotechnology, Dallas, TX), anti-RAGE, anti-PKC (Sigma-Aldrich, Shanghai, China), anti-PKA (Abcam, UK), anti-iNOS (Abcam, UK), anti-SOD1 (Santa Cruz, USA) and anti-actin (Sigma-Aldrich, Shanghai, China). Then HRP-conjugate secondary antibody was utilized and color development achieved with enhanced chemiluminescence (ECL) reagent (EMD Millipore, Billerica, MA, USA).

### Statistical analysis

SPSS13.0 software was used for statistical analysis of all data. Data were presented as mean ± standard deviation (x ± s). And comparisons among multiple groups were performed using ANOVA. P < 0.05 was deemed as statistically significant.

## Results

### Characteristics of pancreatic progenitor cells

**Fig. 1:**
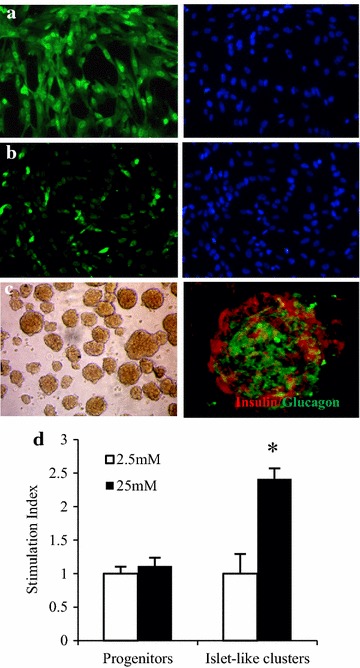
Characteristics of human fetal pancreatic progenitor cells. Human fetal pancreatic progenitor cells were isolated from fetal pancreas at gestational week 8 and maintained in medium containing basic fibroblast growth factor, epidermal growth factor and leukemia inhibitor factor. The expressions of pancreatic endocrine progenitor cell marker Pdx-1 (**a**) and endocrine progenitor markers Ngn 3 (**b**) were monitored by immunofluorescent staining. After induction toward insulin-producing cells, the cells were re-suspended and cultured overnight for forming islet-like cluster. Then immunofluorescent staining was employed for detecting human insulin and glucagon (**c**). Progenitor cell-derived islets were incubated in Krebs–Ringer bicarbonate buffer with 2.5 or 25 mM glucose respectively. And glucose stimulated insulin secretion was measured by ELISA and stimulation index calculated as a ratio of 25 mM group to 2.5 mM group. The experiment was performed thrice with different progenitor cells. Data were represented as mean ± SE, *P < 0.01 versus progenitors at 25 mM (**d**)

Human fetal pancreatic progenitor cells were maintained in medium containing basic fibroblast growth factor, epidermal growth factor and leukemia inhibitor factor. These cells expressed pancreatic progenitor cell marker Pdx-1 (Fig. 1a) and endocrine progenitor marker Ngn 3 (Fig. 1b).

Upon suspending culture with extracellular matrix, differentiated cells formed islet-like clusters (Fig. 1c). Immunofluorescent staining indicated that these progenitor cell-derived islets contained insulin and glucagon-positive cells (Fig. 1c). Moreover, insulin secretion spiked sharply in progenitor cell-derived islets upon hyperglycemic stimulation (Fig. 1d).

### Efficacy of progenitor cell-derived islet transplantation

**Fig. 2:**
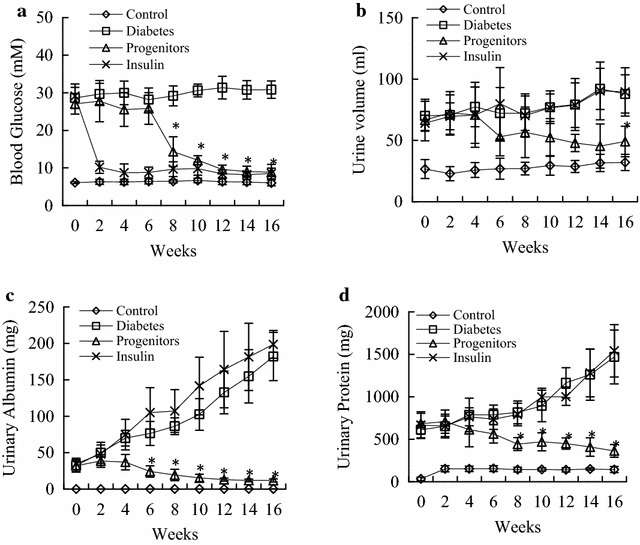
Blood glucose and urinary albumin excretion rate after transplantation in diabetic rats. At different timepoints, the levels of blood glucose, urinary volume, urinary albumin and urinary protein were evaluated. **a** Effects of progenitor cells on glycemic levels in diabetic rats; **b** effects of progenitor cells on urinary volume in diabetic rats; **c** effects of progenitor cells on urinary protein levels in diabetic rats; **d** effects of progenitor cells on urinary albumin levels in diabetic rats. *Control* normal rats, *Diabetes* DN rats treated with saline, *Progenitors* DN rats after transplantation, *Insulin* DN rats treated with insulin. *P < 0.01 progenitor cell group versus diabetic group

Progenitor cell-derived islet transplantation significantly reduced blood glucose in DN rats (Fig. 2a), 24 h urinary volume (Fig. 2b), 24 h urinary albumin (Fig. 2c) and 24 h urinary protein (Fig. 2d). Insulin treatment only significantly reduced blood glucose. And 24 h urinary volume, albumin and protein of insulin-treated rats were elevated and there was no statistical difference from DN rats.

### Renal morphological changes after progenitor cell-derived islet transplantation

Renal hypertrophy is an early feature of DN and its degree is associated with advanced kidney fibrosis. As shown in Additional file 1: Figure S1, regularly shaped control kidneys had smooth surfaces with a reddish-brown color. However, renal morphology was irregular, and there were uneven surfaces with a brownish-yellow color in DN rats. Renal volume was higher in DN rats. And index of renal hypertrophy was greater than that of control rats [(21.10 ± 5.78) vs (5.91 ± 0.79), P < 0.001]. After transplantation, rats had reddish-brown smooth kidneys with volumes falling between volume values of DN group and healthy controls. Renal hypertrophy index was significantly lower in islet transplantation group than that in DN group [(9.75 ± 2.14) vs (21.10 ± 5.78), P < 0.001]. Renal morphology and index of renal hypertrophy of insulin-treated group were not significantly different from those of DN group (P > 0.05).

### Survival of progenitor cell-derived islets in liver

**Fig. 3:**
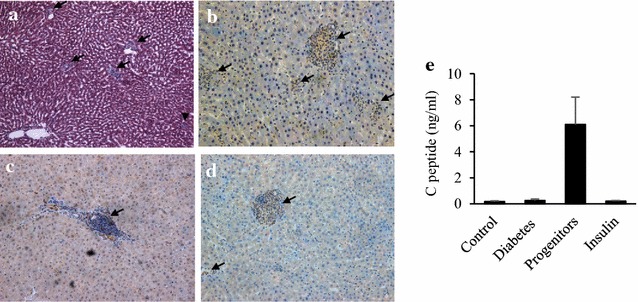
Evaluation of survival of grafted islets in diabetic rat liver. Livers with grafted islets were fixed and sections prepared for HE stain (**a**) (×100), immunohistochemical stains for human mitochondrial antigen (**b**) (×400), human C-peptide (**c**) (×400) and human glucagon (**d**) (×400) respectively. In addition, serum level of human C-peptide increased in progenitor cell group (**e**)

At week 16 post-transplantation, H&E liver staining revealed the presence of transplanted islets in hepatic sinusoids (Fig. 3a). Based upon immunohistochemical staining, these grafts were positive for human mitochondrial antigen and it indicated their human origin (Fig. 3b). And these cells contained human C-peptide-positive (Fig. 3c) and glucagon-positive cells (Fig. 3d), indicating pancreatic endocrine function of these cells. Furthermore, serum level of human C-peptide increased in progenitor cell group as compared with other groups (Fig. 3e). Thus progenitor cell-derived islets survived in liver and had insulin-secreting function.

### Immunogenicity of progenitor cells

Since human fetal pancreatic progenitor cells survived in liver without using immunosuppressant, we analyzed the immunogenicity of fetal pancreatic progenitor cells. As shown in supplemental data, the expression of HLA class I molecules (Additional file 2: Figure S2A) in progenitor cells from 8 to 16th gestational-week increased with development with or without a stimulation of interferon-γ. Also HLA class II molecules (Additional file 2: Figure S2B) showed a similar trend but became slightly up-regulated by interferon-γ. The progenitor cells derived from earlier developmental stage had lower immunogenicity.

By co-culturing rat lymphocytes with progenitor cell lysate, secretion of IL-2 did not increase significantly as compared with positive control group and no statistical difference existed between progenitor cell lysate and negative control groups (Additional file 2: Figure S2C). Moreover, the serum levels of IgG, IgA and IgM from rats after transplantation did not increase significantly as compared with those from rats before transplantation (Additional file 2: Figure S2D).

### Effect of progenitor cells-derived islets on glomerular filtration barrier

**Fig. 4:**
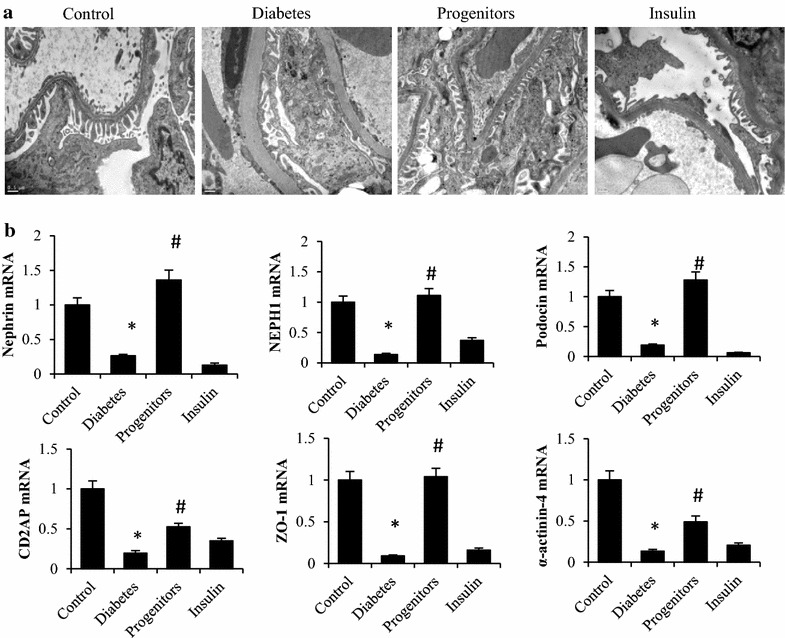
Effects of progenitor cells on the structure of glomerular filtration barrier. After 16-week treatment, rat kidneys were harvested and ultra-structures of glomerular filtration barrier evaluated by transmission electron microscopy (**a**) ×20,000, *Control* normal rats, *Diabetes* DN rats treated with saline, *Progenitors* DN rats after transplantation, *Insulin* DN rats treated with insulin. The mRNA expression of core proteins of GPSD (**b**). *P < 0.01 versus control group, ^#^ *P* < 0.01 versus diabetic group

Alterations of glomerular filtration barrier are the pathophysiological basis of proteinuria in DN. Under electron microscopy (Fig. 4a), podocytes were neatly arranged in healthy controls and the thickness of GBM was 287.60 ± 25.43 nm. In DN rats, podocytes became fused and defective and GMB thickness increased significantly (978.71 ± 63.25 nm). Rats treated with islet cells had fewer fused and defective podocytes than DN group. GMB thickness was 351.17 ± 29.59 nm and it was significantly less than that of DN rats (P < 0.001). Insulin treatment failed to improve podocyte morphology in DN rats. GBM thickness was 876.49 ± 76.27 nm for rats in insulin-treated group and it was not significantly different than that of DN group (P > 0.05).

The core proteins of glomerular podocyte slit diaphragm (GPSD) form the final barrier for plasma protein passing through renal glomerular vasculature. As shown in Fig. 4b, mRNA expressions of glomerular nephrin, *NEPH*-*1*, podocin, *CD2AP*, *ZO*-*1* and α-actinin-4 were significantly lower in DN rats than those in healthy controls (P < 0.001). Progenitor cell-derived islet transplantation restored mRNA expressions of nephrin, *NEPH*-*1*, podocin and *ZO*-*1* to the levels not significantly different from those of controls (P > 0.05). Moreover, the elevated expressions of *CD2AP* and α-actinin-4 were still lower than those in healthy controls. Insulin-treatment did not significantly restore the expressions of genes encoding core proteins of GPSD and the expression of α-actinin-4 was significantly lower than that in DN rats (P < 0.01).

### Effect of islet on glomerulosclerosis

**Fig. 5:**
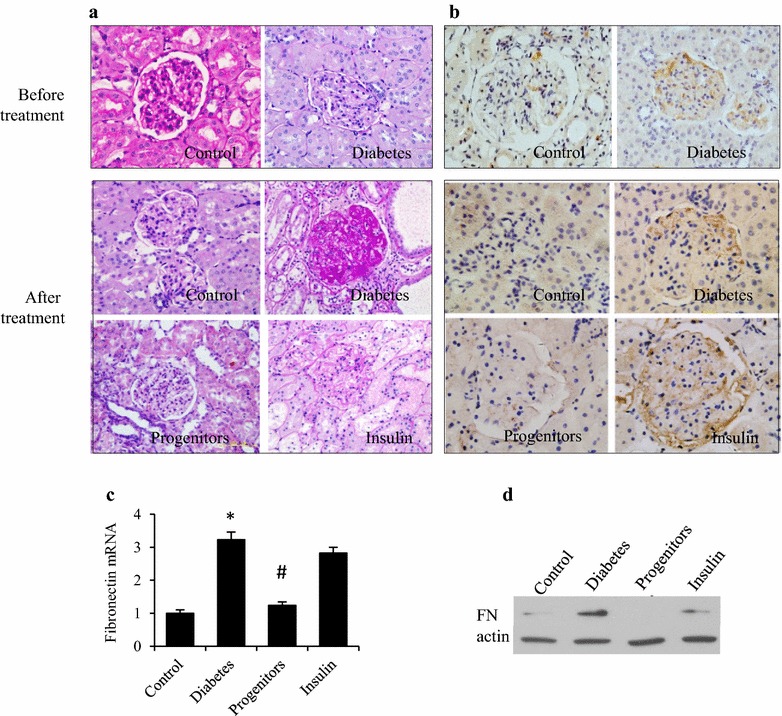
Effects of progenitor cells on glomerulosclerosis in diabetic rats. Kidneys were harvested at week 16 post-transplantation. PAS stain (×200) (**a**) was utilized for observing glomerular morphology and immunohistochemistry stain (**b**), real-time PCR (**c**) and western blot (**d**) were employed for evaluating the expression of fibronectin in glomerular mesangial stroma. *P < 0.01 vs control group, ^#^ *P* < 0.01 versus diabetic group

PAS stain (Fig. 5a) revealed that glomerular capillary loops were thin and transparent in healthy control rats. Glomerular mesangial stroma increased in diabetic rats. At week 16, glomerular mesangial stroma increased significantly in DN rats. And glomerular capillary loops had a deposition of nodular pink, glass-like materials. Islet transplantation significantly reduced the thickness of glomerular capillary loops in DN rats. And insulin treatment showed no improvement. As an important component of glomerular mesangial stroma, FN was expressed more in DN group than controls. It decreased in progenitor cell group but not in insulin group (Fig. 5b). And the differential expression of FN was confirmed by both real-time PCR (Fig. 5c) and western blot (Fig. 5d).

### Mechanism of progenitor cell-derived islet reducing DN

**Fig. 6:**
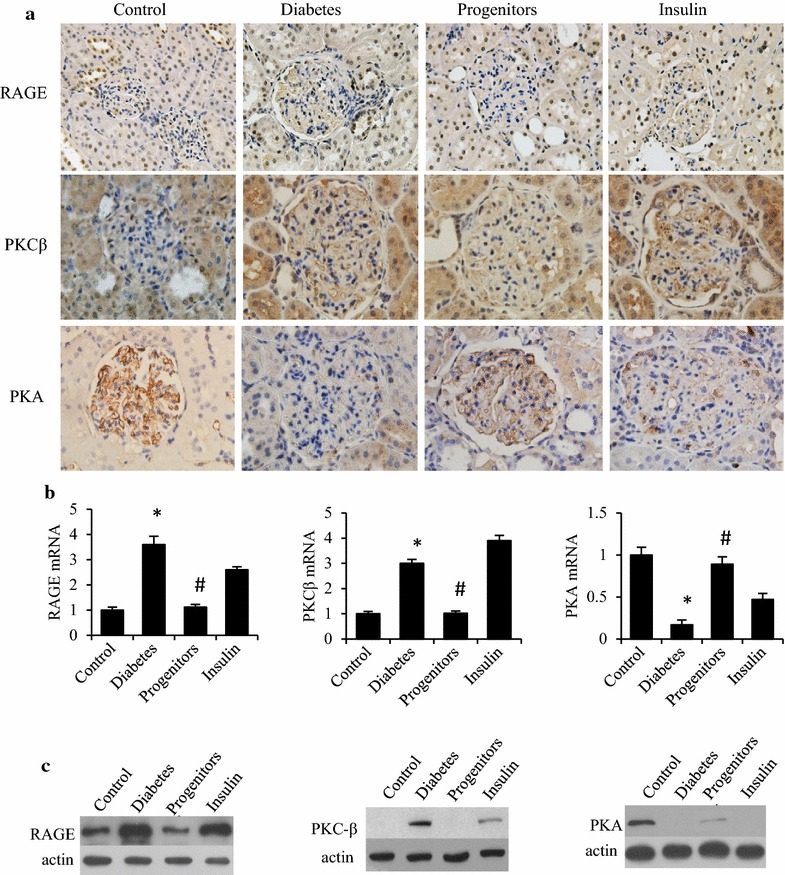
Effects of progenitor cells on RAGE, PKCβ and PKA expression in glomeruli. Kidney cortex tissue was harvested at week 16 after therapy. Then immunohistochemical stain (**a**), real-time PCR (**b**) and western blot (**c**) were employed for evaluating the expressions of RAGE, PKCβ and PKA in glomerular tissue. *P < 0.01 versus control group, ^#^ *P* < 0.01 versus diabetic group

Chronic hyperglycemia leads to the accumulation of AGEs and trigger some signaling pathways. Immunohistochemistry (Fig. 6a) showed that compared to healthy controls, glomerular RAGE and PKC expression in DN rats was significantly higher, whereas PKA expression was significantly down-regulated. Progenitor cells transplantation reduced RAGE accumulation in the glomeruli, down-regulated PKC expression, and upregulated PKA expression in DN rats. Insulin treatment partially reduced RAGE accumulation in the glomeruli of DN rats with relatively weak effects compared with islet transplantation and had no influence on PKC and PKA expression. Similar results were found by real-time PCR (Fig. 6b) and western blot (Fig. 6c).

### Effect of progenitor cell-derived islet on glomerular oxidative stress

**Fig. 7:**
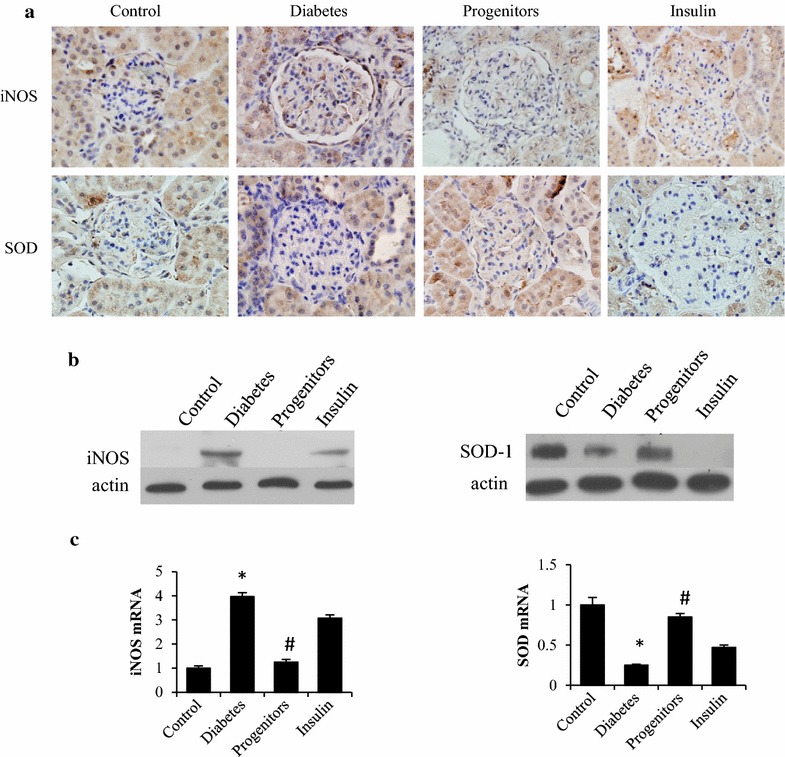
Effects of progenitor cells on iNOS and SOD expression in glomeruli of diabetic rats. Renal cortex tissue was harvested at week 16 post-transplantation. Then immunohistochemical stain ×400 (**a**), real-time PCR (**b**) and western blot (**c**) were employed for evaluating the expressions of iNOS and SOD in glomerular tissue. *P < 0.01 versus control group, ^#^ *P* < 0.01 versus diabetic group

To evaluate the oxidative stress level of glomeruli, two important enzymes were measured. Immunohistochemistry (Fig. 7a) demonstrated that glomerular iNOS expression was significantly higher in DN rats while glomerular expression of SOD1 was significantly lower than controls. Islet transplantation significantly reduced iNOS expression and increased SOD1 expression in DN rats. However, insulin treatment failed to alter the expressions of glomerular iNOS and SOD in DN rats. And glomerular iNOS and SOD expression was similar in insulin-treated rats to untreated DN rats. Real-time PCR (Fig. 7b) and western blot (Fig. 7c) data agreed with the findings of immunohistochemistry.

## Discussion

Insulin producing cells derived from progenitor cells were reported effectively in reducing blood glucose in diabetic animals [14–16]. In this study, progenitor cell-derived islet transplantation improved significantly not only blood glucose but also DN in DN animals.

Fetal pancreatic progenitor cells were isolated from 8th gestational week. It was reported that first-trimester human fetal pancreas had lower immunogenicity than second-trimester pancreas [17]. The immunogenicity of pancreatic progenitor cells and islet-like cell clusters from first-trimester was much lower than that of first-trimester human fetal pancreas tissue [18]. Moreover, these cells had a distinctively lower MHC I & II expression relative to second-trimester pancreatic progenitor cells, even after IFNγ challenge [18]. Our results indicated that progenitor cells isolated from 8th gestational week exhibited low immunogenicity in vitro and in vivo. Transplanted progenitor cell-derived islets could survive longer in liver and effectively reduced DN without immunosuppressants.

As an early sign of DN, microalbuminuria gradually develops into refractory proteinuria and ultimately renal failure [19, 20]. Reduction of urinary albumin is a marker for improved DN [21]. Our data show that progenitor cell-derived islets could survive in liver and urinary protein and albumin decreased in diabetic rats.

Structural and functional changes of glomerular filtration barrier, composed of podocytes, GBM and endothelial cells, are the pathophysiological basis of proteinuria in DN. Adjacent processes between podocytes alternate with each other and form a 30–40-nm slit covered with a 4–6 nm membrane layer of GPSD. This is the final barrier for plasma protein passing through renal glomerular vasculature and maintaining the structural and functional integrity of glomerular filtration barrier [22]. Progenitor cell-derived islets could reduce GBM thickness in diabetic rats and it explained a lower level of urinary albumin.

Mostly distributed in glomerular mesangial matrix, FN is a major non-collagenous glycoprotein within glomerular ECM. As a result, any change of FN fully reflects in glomerular ECM. Islet transplantation inhibited the glomerular accumulation of FN in DN rats. Progenitor cell-derived islet could up-regulate the expression of GPSD molecules. Thus progenitor cell-derived islets might reverse DN through improving the structural integrity of glomerular podocytes, GPSD and GBM.

As confirmed by in vivo experiments and clinical trials, AGEs significantly increased in types I & II DM. And AGEs interact with its receptor RAGE for promoting the pathogenesis of DN. A combination of AGEs and RAGE promotes ROS generation, activates the renin-angiotensin system and interacts with such signaling molecules as microtubule-affinity-regulating-kinase (MARK), nuclear factor-kappa B (NF-κB) and PKC. And PKC family includes at least 12 subunits [23] and PKC-β subunit is a major subunit involved in pathological changes of DN [24]. Our previous study found that bonding of AGEs-RAGE contributed to DN by an up-regulation of PKC-β and a down-regulation of PKA, thereby promoting DN via oxidative stress [4]. Moreover, the glomerular accumulation of FN was also correlated with an up-regulation of RAGE and a down-regulation of PKA [25]. In this study, progenitor cell-derived islets could reduce DN by lowering RAGE and PKC but increasing PKA in glomeruli.

Previous studies suggest that PKC and PKA signaling pathways are closely correlated with oxidative stress. Two major causes of oxidative stress are an excessive generation of ROS and insufficient antioxidants. And iNOS expression was higher and SOD1 expression was lower in diabetic rats than controls. Progenitor cell-derived islet transplantation reduced oxidative stress, lowered RAGE and PKC and elevated PKA in DN rats.

Although insulin treatment lowered and maintained stable blood glucose in our rat model, its therapeutic effect was insignificant, suggesting that simple control of blood glucose failed to restore structural and functional renal lesions in DN rats.

The efficacy of progenitor cell-derived islets may be due to insulin. Immunohistochemical staining showed that progenitor cell-derived islets secreted insulin, C-peptide and human glucagon in liver. The findings were similar to those of previous reports [14, 26]. And C-peptide was efficacious for diabetic microangiopathy, especially DN [27–30]. Dosing of C-peptide could significantly improve renal size, morphology and function in DN rats [25, 31–33]. In humans, short and long-term treatments of C-peptide affected renal regulatory and physiological functions in type I DM [34–36]. With a restoration of endogenous insulin after transplantation, mechanisms behind C-peptide-mediated microvascular improvements may be due to specific binding of G-protein coupled receptors to renal tubular and mesangial cells [37]. Besides C-peptide, glucagon secretion from A-cells of pancreatic islets offered supports for beta cell function. Excessive digestion of isolated islets depleted alpha cells and impaired islet function after transplantation. However, it is unclear whether or not glucagon is involved in other mechanisms of direct improvements in DN rats.

Beside insulin replacement, indirect effects of pancreatic progenitor cannot be ruled out in this study. For example, it may have immune modulation and paracrine action on liver. Recent years, immune modulation of several kinds of stem cells is accumulated. A phase 1/phase 2 study showed that T2D patients achieve improved metabolic control and reduced inflammation markers after receiving autologous mononuclear cells which briefly co-cultures with adherent cord blood-derived multipotent stem cells. The reason is stem cells modulating immune function of monocytes and balancing Th1/Th2/Th3 cytokine production [38]. Mesenchymal stem cell was also well known by the moderating immune response of type 1 diabetes [39]. In addition, allogeneic adipose-derived mesenchymal stem cell (ADMSCs) was reported to ameliorate experimental autoimmune diabetes via downregulation of the CD4(+) Th1-biased immune response and expansion of regulatory T cells (Tregs) in the pancreatic lymph nodes. In vitro, ADMSCs induced the expansion/proliferation of Tregs in a cell contact-dependent manner mediated by programmed death ligand 1 [40]. Although there is no data about the effect of pancreatic progenitors on Tregs, it is valuable to do further investigation.

We suppose that one of the other mechanisms of pancreatic progenitor cells maybe ameliorates insulin resistance by paracrine effect. As shown in MSC, glucose uptake in peripheral tissues, including skeletal muscle and adipose tissue, was elevated in MSC-treated mice. Furthermore, enhanced glucose uptake in these tissues was associated with improved insulin signaling as assessed by Akt phosphorylation and the expression of GLUT-4 [41]. In summary, pancreatic progenitor cells may improve DN by multi-ways.

## Conclusions

Transplantation of progenitor cell-derived islet may effectively improve the glomerular filtration barrier structure and reverse DN by modulating the glomerular expressions of PKC and PKA and altering the oxidative stress level.

## Additional files

**Additional file 1: Figure S1.** Effect of pancreatic endocrine progenitor cells on renal morphology of diabetic rats. DN rats were transplanted with progenitor cell-derived islets or treated with insulin for 16 weeks. Then renal morphology was observed (A) and renal index calculated (B). *P<0.01 versus control group, ^#^ *P*<0.01 versus diabetic group.

**Additional file 2: Figure S2.** Immunogenicity of human fetal pancreatic progenitor cells. To evaluate the immunogenicity of human fetal pancreatic derived progenitor cells, the expressions of HLA classes I (A) and II (B) molecules were compared by flow cytometry between progenitor cells originating from different developmental stages. The results were expressed as mean fluorescence intensity. Furthermore, rat PBMCs were incubated with progenitor cells lysate and secretion of IL-2 by PBMCs was measured by ELISA (C). In addition, serum level of anti-human IgG in grafted rats was detected at week 16 post-transplantation (D). All figures represented one of three independent experiments and data were shown as mean ± SD.

## Abbreviations

DN: diabetic nephropathy
AGEs: advanced glycation end products
RAGE: receptor of AGEs
PKC: protein kinase C
PKA: protein kinase A
GLP-1: glucagon-like peptide 1
STZ: streptozotocin
HLA: human leukocyte antigen
PMA: phorbol**-**12-myristate-13-acetate
PHA: phytohemagglutnin
PAS: periodic acid-Schiff
FN: fibronectin
iNOS: inducible nitric oxide synthase
SOD: superoxide dismutase
GBM: glomerular basement membranes
