# Supplementary material for: Transplantation of human fetal pancreatic progenitor cells ameliorates renal injury in streptozotocin-induced diabetic nephropathy
Source: J Transl Med. 2017 Jun 27;15:147. doi: 10.1186/s12967-017-1253-1 (PMC5488369; doi:10.1186/s12967-017-1253-1)
Supplement: Supplementary file 1 — Additional file 1: Figure S1. Effect of pancreatic endocrine progenitor cells on renal morphology of diabetic rats. DN rats were transplanted with progenitor cell-derived islets or treated with insulin for 16 weeks. Then renal morphology was observed (A) and renal index calculated (B). *P<0.01 versus control group, # P<0.01 versus diabetic group. [file 12967_2017_1253_MOESM1_ESM.pptx]

## Slide 1
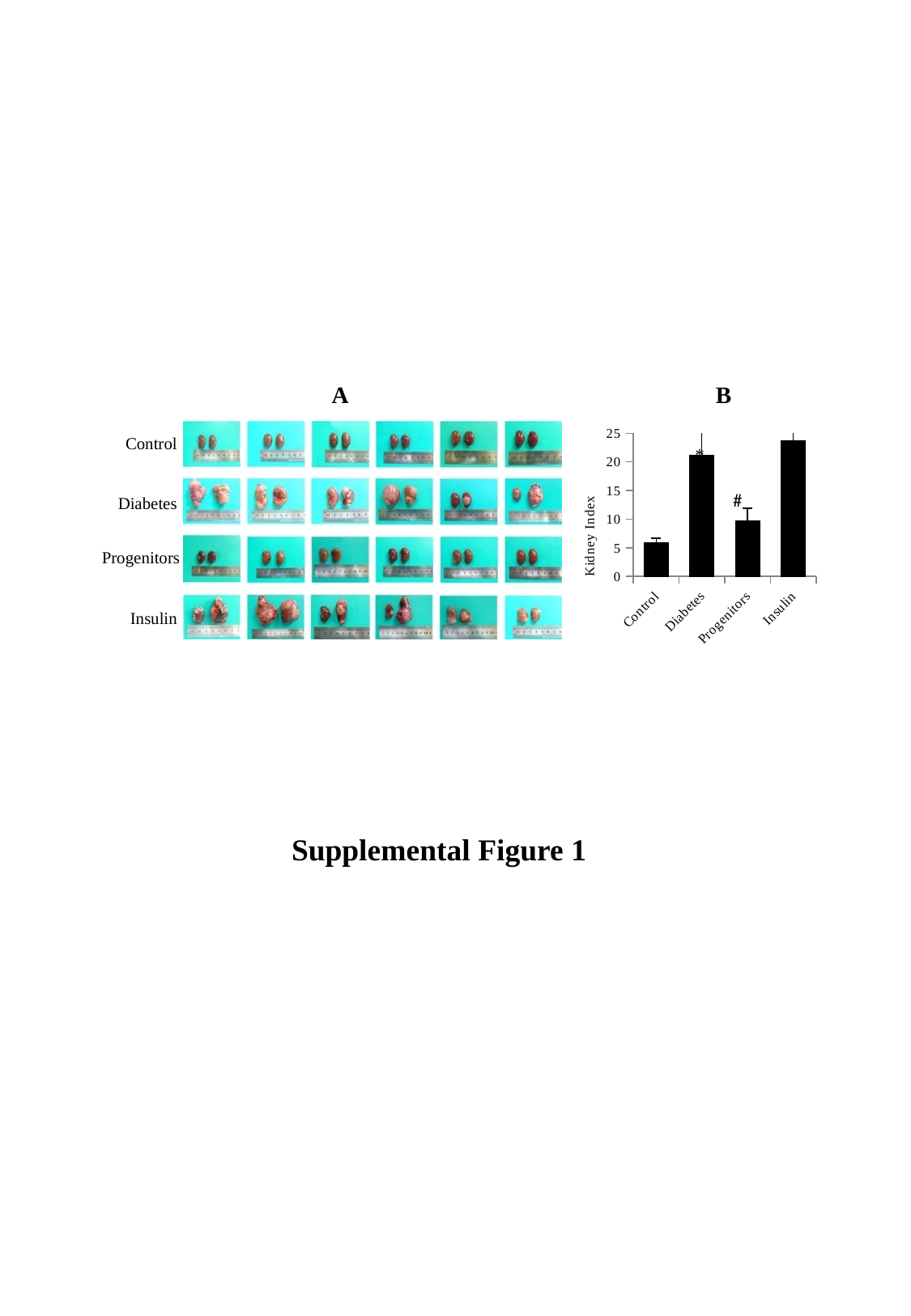

A
B
Control
Diabetes
Progenitors
Insulin
### Chart
| Category | |
|---|---|
| Control | 5.9 |
| Diabetes | 21.1 |
| Progenitors | 9.75 |
| Insulin | 23.77 |Supplemental Figure 1
