# Supplementary material for: Transplantation of human fetal pancreatic progenitor cells ameliorates renal injury in streptozotocin-induced diabetic nephropathy
Source: J Transl Med. 2017 Jun 27;15:147. doi: 10.1186/s12967-017-1253-1 (PMC5488369; doi:10.1186/s12967-017-1253-1)
Supplement: Supplementary file 2 — Additional file 2: Figure S2. Immunogenicity of human fetal pancreatic progenitor cells. To evaluate the immunogenicity of human fetal pancreatic derived progenitor cells, the expressions of HLA classes I (A) and II (B) molecules were compared by flow cytometry between progenitor cells originating from different developmental stages. The results were expressed as mean fluorescence intensity. Furthermore, rat PBMCs were incubated with progenitor cells lysate and secretion of IL-2 by PBMCs was measured by ELISA (C). In addition, serum level of anti-human IgG in grafted rats was detected at week 16 post-transplantation (D). All figures represented one of three independent experiments and data were shown as mean ± SD. [file 12967_2017_1253_MOESM2_ESM.pptx]

## Slide 1
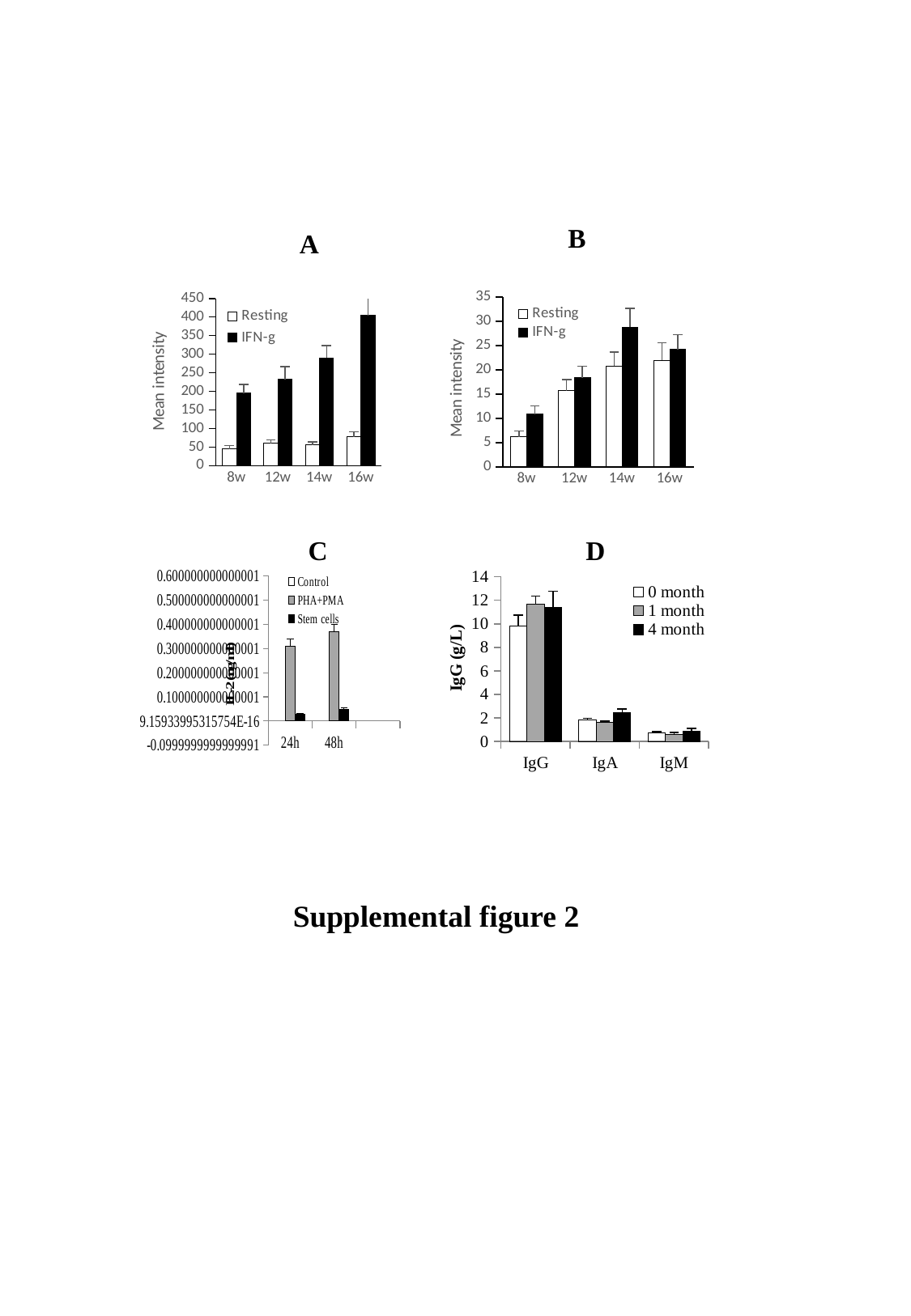

B
A
### Chart
| Category | Resting | IFN-g |
|---|---|---|
| 8w | 6.2 | 10.9 |
| 12w | 15.7 | 18.4 |
| 14w | 20.8 | 28.8 |
| 16w | 22.0 | 24.2 |
### Chart
| Category | Resting | IFN-g |
|---|---|---|
| 8w | 45.2 | 194.9 |
| 12w | 60.0 | 232.4 |
| 14w | 56.2 | 289.8 |
| 16w | 77.6 | 404.2 |C
D
### Chart
| Category | 0 month | 1 month | 4 month |
|---|---|---|---|
| IgG | 9.82 | 11.7 | 11.4 |
| IgA | 1.8 | 1.6 | 2.42 |
| IgM | 0.700000000000001 | 0.620000000000002 | 0.89 |
### Chart
| Category | Control | PHA+PMA | Stem cells |
|---|---|---|---|
| 24h | 0.0298 | 0.3081 | 0.0282 |
| 48h | 0.0503 | 0.368800000000001 | 0.0485 |Supplemental figure 2
